# COVID-19 vaccination for children with pulmonary hypertension: efficacy, safety and reasons for opting against vaccination

**DOI:** 10.3389/fped.2023.1259753

**Published:** 2023-10-04

**Authors:** Zeying Zhang, Dan Wang, Wanyun Zuo, Xun Wang, Fan Yang, Haiyan Luo, Zhenghui Xiao, Qiming Liu, Yunbin Xiao

**Affiliations:** ^1^ Department of Cardiology, The Second Xiangya Hospital of Central South University, Changsha, China; ^2^ Department of Cardiology, Hunan Children’s Hospital, Changsha, China; ^3^ Department of Hematology, The Second Xiangya Hospital of Central South University, Changsha, China

**Keywords:** pulmonary hypertension, COVID-19, vaccination, hesitation, adverse effects

## Abstract

**Objective:**

To determine the reasons why pulmonary hypertension (PH) children refused vaccination against COVID-19, evaluate the safety and efficacy of COVID-19 vaccine in PH children.

**Study design:**

This retrospective cohort study included congenital heart disease-associated pulmonary arterial hypertension (CHD-PAH) and bronchopulmonary dysplasia associated PH (BPD-PH) children who were divided into vaccinated group and non-vaccinated group. Univariate logistic regression analysis and multivariate logistic regression analysis were conducted to explore the reasons why PH children refused COVID-19 vaccine. Then, the prevalence, the number of symptoms, and the severity of COVID-19 disease were compared between the vaccinated and unvaccinated groups.

**Result:**

We included 73 children and 61 children (83.6%) were unvaccinated. The main reasons for not being vaccinated were fear of worsening of existing diseases (31%). Age < 36 months (RR: 0.012; *P* < 0.001) and the presence of comorbidities (RR = 0.06; *P* = 0.023) were risk factors influencing willingness to vaccinate. The most common adverse events (AEs) were injection site pain (29.6%). COVID-19 vaccines are safe for PH children. The prevalence of COVID-19 disease decreased in PH children after vaccination (RR = 0.51; *P* = 0.009). 1 month after negative nucleic acid test or negative antigen test, PH children in the vaccinated group had fewer symptoms (*P* = 0.049).

**Conclusions:**

The vaccination rate of COVID-19 vaccine is low in CHD-PAH and BPD-PH children while COVID-19 vaccines are safe. Vaccination can reduce the prevalence of COVID-19 disease and the number of symptoms 1 month after negative nucleic acid or antigen tests.

## 1. Introduction

In the context of COVID-19 global pandemic, patients with cardiovascular and pulmonary comorbidities are at high risk of adverse COVID-19 disease outcomes (1–3). Underlying chronic respiratory disease increased COVID-19 disease mortality (4). PH is a pathophysiological state of abnormally elevated pulmonary blood pressure and pulmonary vascular resistance, which eventually leads to right heart hypertrophy, right heart failure and death (5). The hemodynamic definition of PH is: mean pulmonary artery pressure (mPAP) > 20 mmHg measured by right heart catheterization at sea level and at rest (6). Referring to the 6th WSPH (world symposium on pulmonary hypertension) guidelines in 2018 (7), the clinical classification of PH is: (1) pulmonary arterial hypertension, (2) PH caused by left heart disease; (3) PH caused by pulmonary disease and/or hypoxia; (4) PH caused by pulmonary artery obstructive disease; (5) unknown mechanism and/or multiple factor-induced PH. As with other cardiorespiratory disorders, patients with PH are at particularly high risk for clinical exacerbations associated with respiratory infections (8, 9). Reciprocally, COVID-19 disease can also cause short-term or long-term adverse cardiovascular effects (10). COVID-19 disease, as a severe pulmonary infection, causes right ventricular systolic dysfunction (11, 12). This may be detrimental in patients with PH and chronic right ventricular dysfunction. Indeed, the incidence of COVID-19 disease in PH patients was similar to that of the general population, but disease severity and prognosis were much worse in these patients (9, 13).

COVID-19 vaccination not only enhances the protective effect of the general population and comorbidities against COVID-19 infection and COVID-19 severity, but also reduces mortality (14–16). COVID-19 vaccination reduces the prevalence of COVID-19 disease in adults with PH (17). COVID-19 vaccines are advocated in adult patients with PH. The global COVID-19 pandemic is of particular concern to PH children due to immature immune systems and developmental comorbidities (18). However, data on the effectiveness and safety of COVID-19 vaccination in PH children are unclear. The purpose of this single-center retrospective cohort study was to determine COVID-19 vaccination status and explore the effectiveness and safety of vaccination in PH children.

## 2. Materials and methods

### 2.1. Study group

This retrospective observational study was conducted at a single PH center in China. Inclusion criteria: (1) hospitalization in Hunan Children's Hospital since May 2022; (2) age < 18 years; (3) diagnosed with PH according to the diagnostic criteria of current guidelines for PH (6). Exclusion criteria: children who had been infected with COVID-19 before the vaccination date. The date of confirmed COVID-19 disease was determined as the date of the first positive nucleic acid test or antigen test. All children or their guardians gave written consent to participate in the study. In accordance with the Declaration of Helsinki, the Ethics Committee of Hunan Children's Hospital approved the research protocol (KS2023-76). Written informed consent has been obtained from the parent or guardian of the child subject of the investigation and, where appropriate, the consent of the subject himself. The manuscript does not contain natural and identifiable information, including the patient's image, name, or hospital number.

Information about patient demographics, PH-related information, and COVID-19 disease-related information was obtained from the children's medical records (Table 1). Through the designed questionnaire, the data of the children and their families about COVID-19 disease were investigated, including vaccination (vaccination date, dose, vaccine manufacturer and local or systemic AEs after vaccination), prevalence, symptoms, severity and duration, COVID-19 disease -related treatment measures, and symptoms pessenting after 1 month when nucleic acid test or antigen test turn negative. Vaccination doses were calculated on the date of the questionnaire. The severity of PH in children is determined according to mPAP: (1) Mild: 20–40 mmHg; (2) Moderate: 40–70 mmHg; (3) Severe: >70 mmHg. The risk classification criteria were determined in accordance with the 2019 Guidelines for the Diagnosis and Treatment of PH in Children (6). Children who could not be included in the lower or higher risk PH subgroup were placed in the intermediate-risk subgroup. Evidence for the diagnosis of COVID-19 disease (19): (1) Etiological test: take nasal, oropharyngeal swabs or lower respiratory tract secretions for COVID-19 nucleic acid test, and the result is positive; (2) Serological test: IgM of COVID-19 The antibody test was positive. Clinical classification of COVID-19 disease (19): (1) Mild: upper respiratory tract infection symptoms are the main manifestations, such as dry throat, sore throat, cough, fever, etc. (2) Moderate: persistent high fever >3 days or (and) cough, shortness of breath, etc., but respiratory rate (RR) < 30 breaths/min, oxygen saturation >93% when inhaling air in a resting state. Imaging examinations showed characteristic pulmonary manifestations of COVID-19 disease. (3) Severe: ultra-high fever or persistent high fever for more than 3 days; shortness of breath (<2 months old, RR ≥ 60 times/min; 2–12 months old, RR ≥ 50 times/min; 1–5 years old, RR ≥ 40 times/min /min; >5 years old, RR ≥ 30 times/min); in resting state, oxygen saturation ≤93% when inhaling air; nasal flapping, three-concave sign or wheezing; disturbance of consciousness or convulsions; refusal to eat or feed difficult, with signs of dehydration. 4) Critical: One of the following situations occurs. Respiratory failure requiring mechanical ventilation; shock; combined with other organ failure requiring intensive care unit (ICU) treatment. The severity of COVID-19 disease in this study is graded using 4 levels of serial numbers: (1) Not hospitalization; (2) Hospitalization without oxygen; (3) Hospitalization with oxygen; (4) ICU. Multisystem inflammatory syndrome in children (MISC) refers to persistent fever and a range of symptoms, including hypotension, involvement of multiple organs such as the heart, gastrointestinal, haematology, dermatology, and neurology, and elevated markers of inflammation. Pulmonary imaging changes refer to multiple small patchy shadows and interstitial changes or multiple ground-glass opacities by chest X-ray or CT scan. Myocardial damage refers to the increase of blood myocardial enzymes.

**Table 1:** Characteristics of included patients groups according to their decisions about vaccination against COVID-19.

	Total study group *n* (%) or Median (IQR)	Vaccinated *n* (%) or Median (IQR)	Unvaccinated *n* (%) or Median (IQR)	*P-*value
Number of patients	73 (100)	12 (16.4)	61 (83.6)	
Age, months	25.6 (37.9)	82.7 (48.4)	22.6 (15.5)	<0.001*
Sex, male	48 (64.9)	8 (66.7)	40 (65.6)	1.000
BMI, kg/m^2^	14.8 (2.6)	14.7 (4.1)	14.8 (2.7)	0.794
Place of residence, city	49 (67.1)	7 (58.3)	42 (68.9)	0.709
Duration of PH, months	17.0 (18.5)	41.5 (35.0)	15.0 (15.0)	0.004*
PH clinical group				0.112
CHD-PAH	25 (34.2)	7 (58.3)	18 (29.5)	
BPD-PH	48 (65.8)	5 (41.7)	43 (70.5)	
Severity of PH				0.759
Mild	23 (31.5)	3 (25.0)	20 (32.8)	
Moderate	26 (35.6)	4 (33.3)	22 (36.1)	
Severe	24 (32.9)	5 (41.7)	19 (31.1)	
Risk classification of PH				0.304
Lower	16 (21.9)	3 (25.0)	13 (21.3)	
Intermediate	38 (52.1)	8 (66.7)	30 (49.2)	
Higher	19 (26.0)	1 (8.3)	18 (29.5)	
Target drugs				0.826
None	4 (6.6)	0 (0)	4 (6.6)	
Monotherapy	25 (34.2)	4 (33.3)	21 (34.4)	
Dual therapy	38 (52.1)	7 (58.3)	31 (50.8)	
Triple therapy	6 (8.2)	1 (8.3)	5 (8.2)	
Other drugs	67 (90.4)	12 (100.0)	55 (90.2)	0.581
Digoxin	48 (65.8)	4 (33.3)	44 (72.1)	0.024**
Furosemide	24 (32.9)	5 (41.7)	19 (31.1)	0.709
Spironolactone	27 (37.0)	6 (50.0)	21 (34.4)	0.307
Potassium chloride	13 (17.8)	3 (25.0)	10 (16.1)	0.764
Chalybeate	32 (43.8)	3 (25.0)	29 (47.5)	0.263
Comorbid conditions	26 (35.6)	1 (8.3)	25 (40.1)	0.046**
Hypothyroidism	5 (19.2)	0 (0)	5 (8.2)	0.583
Asthma	8 (30.8)	1 (8.3)	7 (11.5)	>0.9999
Thrombocytopenia	2 (7.7)	0 (0)	2 (3.3)	>0.9999
Trisomy 21 syndrome	1 (3.8)	0 (0)	1 (1.6)	>0.9999
Food allergies	5 (19.2)	0 (0)	5 (8.2)	0.583
Secondary epilepsy	1 (3.8)	0 (0)	1 (1.6)	>0.9999
Adenoid hypertrophy	2 (7.7)	0 (0)	2 (3.3)	>0.9999
Pharyngomalacia	1 (3.8)	0 (0)	1 (1.6)	>0.9999
Growth retardation	1 (3.8)	0 (0)	1 (1.6)	>0.9999
PH family history	2 (7.7)	1 (8.3)	1 (1.6)	0.740
Family COVID-19 disease	62 (84.9)	9 (75.0)	53 (86.9)	0.541

BMI, body mass index; PH, pulmonary hypertension; monotherapy: bosentan or sildenafil; dual therapy: bosentan + sildenafil or bosentan + tadalafil; triple therapy: bosentan + sildenafil + treprostinil or bosentan + tadalafil + treprostinil.

* Mann–Whitney *U*-test.

** Chi-square test or chi-square correction test.

### 2.2. Statistical analysis

Standard descriptive statistics were used to present the demographic characteristics of the individuals included in the study cohort. Continuous data are expressed as median [interquartile range (IQR)] and categorical data as percentages. The included patients were divided into 2 groups, namely vaccinated and non-vaccinated, according to whether they were vaccinated or not. Demographic, clinical features, and COVID-19 related data were compared between vaccinated and non-vaccinated groups, either by chi-square test, Fisher exact test, or rank-sum test (Mann–Whitney test). Univariate logistic regression analysis and multivariate logistic regression analysis methods were carried out to analyze the risk factors of vaccination intention. Factors with a *P*-value of <0.1 in univariate logistics regression analysis were included in the multivariate logistics regression analysis model. All statistical analyses were performed using SPSS 25.0 software (IBM). A *P* value less than 0.05 is considered statistically significant.

## 3. Results

### 3.1. Study groups

A total of 73 PH children were included in the study. The median age of participants was 25.6 months (IQR 37.9 months). Males predominate (48 cases, 67.9%). The study group included 25 (34.2%) children with CHD-PAH and 48 (65.8%) children with BPD-PH. 12 children (16.4%) were vaccinated against COVID-19, and 61 patients (83.6%) did not get vaccinated. All vaccinated patients completed a two-dose inactivated vaccines schedule. In the vaccinated group, 75 patients (58.3%) had CHD-PAH and 5 (41.7%) had BPD-PH. In the unvaccinated group, 18 (29.5%) patients had CHD-PAH and 43 (70.5%) had BPD-PH. The age of the vaccinated group was greater than that of the unvaccinated group (median age 82.4 months, IRQ: 48.4 years vs. 22.6 months, IQR: 15.5 months; *P* = 0.000). There was no significant difference in the proportion of male patients between the two groups (vaccinated group: 8 cases, 66.7%; unvaccinated group: 40 cases, 65.6%). More people in the unvaccinated group used digoxin than in the vaccinated group (the vaccinated group: 4, 33.3% vs. unvaccinated group: 44, 72.1%, *P* = 0.024). The number of included patients with comorbidities in the vaccinated group was smaller than that in the unvaccinated group (the vaccinated group: 1, 8.3% vs. the unvaccinated group: 25, 40.1%; *P* = 0.046). Other clinical factors (BMI, place of residence, clinical group of PH, severity and risk degree of PH, PH-targeted therapy, family history of PH) were not statistically significant between the vaccinated and unvaccinated groups. The baseline characteristics of the study groups and individual subgroups are shown in Table 1.

### 3.2. Reasons for not being vaccinated

**Figure 1:**
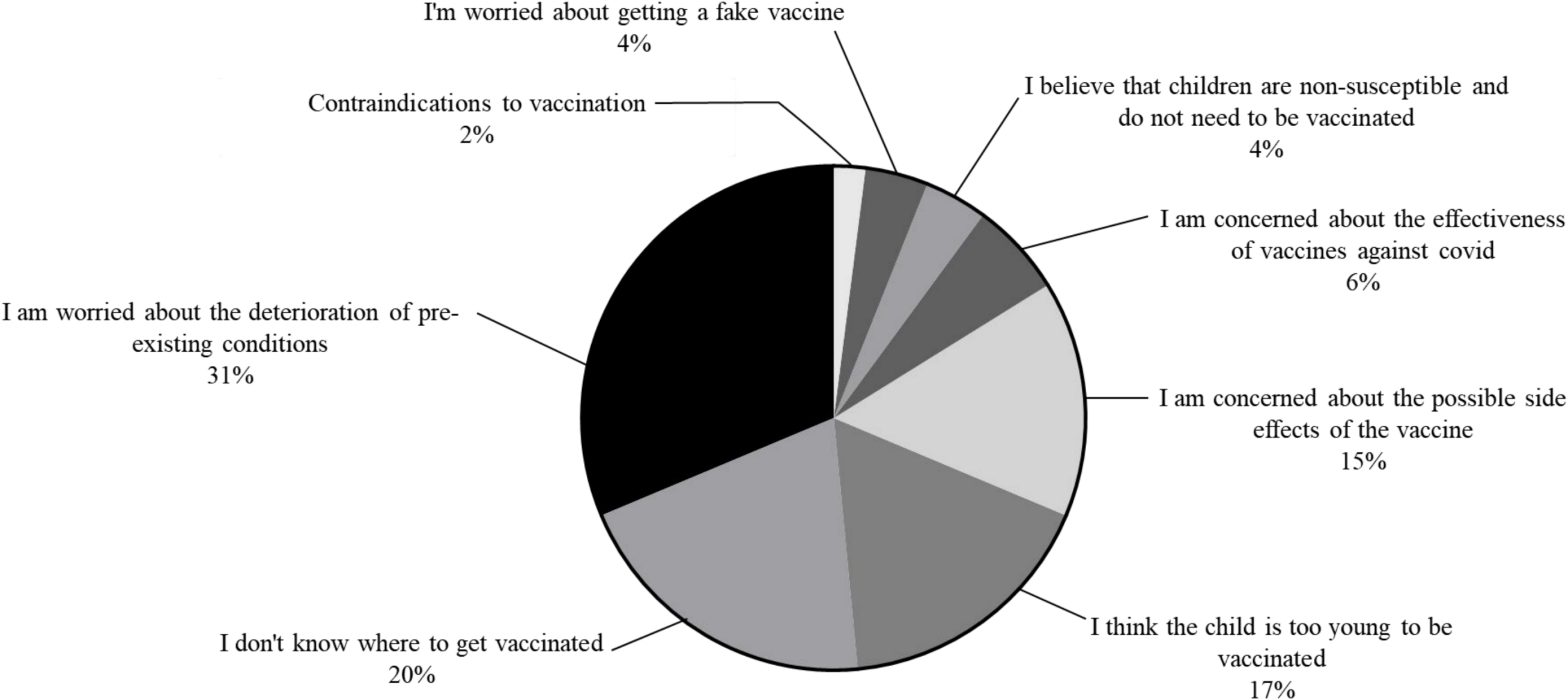
The reason for the unwillingness of children patients with PH to vaccinate against COVID-19.

61 (86.3%) PH children with refused to receive COVID-19 vaccine. The main reason for refusing to receive the COVID-19 vaccine was the fear of worsening the child's pre-existing conditions (*n* = 19; 31% of unvaccinated patients; Figure 1). The second reason is that there is no COVID-19 vaccine supply at a nearby medical institution or the address of the medical institution that supplies the COVID-19 vaccine is not known, although the child's family wants to be vaccinated (*n* = 12; 20% of patients). Third, the child's family believes that the age is too young to be vaccinated against COVID-19 disease (*n* = 10; 17%). Fourth, family members were concerned about possible AEs after vaccination (*n* = 9; 15%). The fifth reason is that COVID-19 vaccines are not considered effective in preventing COVID-19 infection (*n* = 4; 6%). Sixth, the child's family believes that the child is not susceptible to the COVID-19 (*n* = 3; 4%). The seventh reason was the fear of receiving fake vaccines (*n* = 3; 4%). The last reason was contraindications to vaccination (*n* = 1; 2%). Clinical and demographic factors that may influence the decision not to vaccinate are attempted to identify. Age < 36 months, PH clinical group of PAH, PH duration, digoxin use, and presence of comorbidities in univariate logistics regression analysis were included in multivariate logistics regression analysis. In multivariate logistic regression analysis, age < 36.0 months (RR = 0.012, 95% CI: 0.001–0.016; *P* = 0.000) decreased willingness to receive COVID-19 vaccines. PH children with comorbidities (RR = 0.06, 95% CI: 0.01–0.68, *P* = 0.023) were less willing to receive COVID-19 vaccine. The results of the logistics regression analysis are shown in Table 2.

**Table 2:** Impact of demographic and clinical factors on the willingness to vaccinate against COVID-19 in PH children patients: results of the univariate and multivariate regression analyses.

	Univariate analysis RR (95%CI)	*P*-value (LR)	Multivariate analysis RR (95%CI)	*P-*value (Wald, Back)
Age < 36 months	0.02 (0.00–0.15)	<0.001*	0.012 (0.001–0.016)	<0.001**
Male gender	1.0 (0.3–3.9)	0.942		
BMI	1.0 (0.9–1.2)	0.883		
Place of residence, city	1.4 (0.2–12.8)	0.751		
CHD-PAH	3.3 (0.9–11.9)	0.063*		
Duration of PH	1.1 (1.1–1.2)	0.004*		
PAH family history	0.2 (0.0–3.2)	0.243		
Severity of PH (relative to mild)		0.762		
Moderate	0.6 (0.1–2.7)	0.481		
Severe	0.7 (0.2–2.9)	0.617		
PH risk classification (relative to mild)		0.362		
Moderate	4.2 (0.4–44.6)	0.240		
Severe	4.8 (0.6–41.6)	0.155		
Target drugs (relative to none target drug)		0.996		
Monotherapy	–	0.999		
Dual therapy	1.0 (0.1–10.5)	0.968		
Triple therapy	1.1 (0.1–11.2)	0.918		
Use of other drugs	–	0.999		
Digoxin	0.2 (0.1–0.7)	0.015*		
Presence of comorbid conditions	0.1 (0.0–1.1)	0.059*	0.063 (0.01–0.68)	0.023**
Family members COVID-19 disease	2.2 (0.5–9.9)	0.302		

BMI, body mass index; PAH, pulmonary arterial hypertension; PH, pulmonary hypertension; monotherapy: bosentan or sildenafil; dual therapy: bosentan + sildenafil or bosentan + tadalafil; triple therapy: bosentan + sildenafil + treprostinil or bosentan + tadalafil + treprostinil; Use of other drugs: the use of anyone drug of digoxin, furosemide, spironolactone, potassium chloride and chalybeate; Presence of comorbid conditions: the occurrence of anyone conditions of hypothyroidism, asthma, thrombocytopenia, trisomy 21 syndrome, food allergies, secondary epilepsy, adenoid hypertrophy, pharyngomalacia, and growth retardation.

* *P* < 0.1.

** *P* < 0.05.

### 3.3. Vaccination AEs

**Figure 2:**
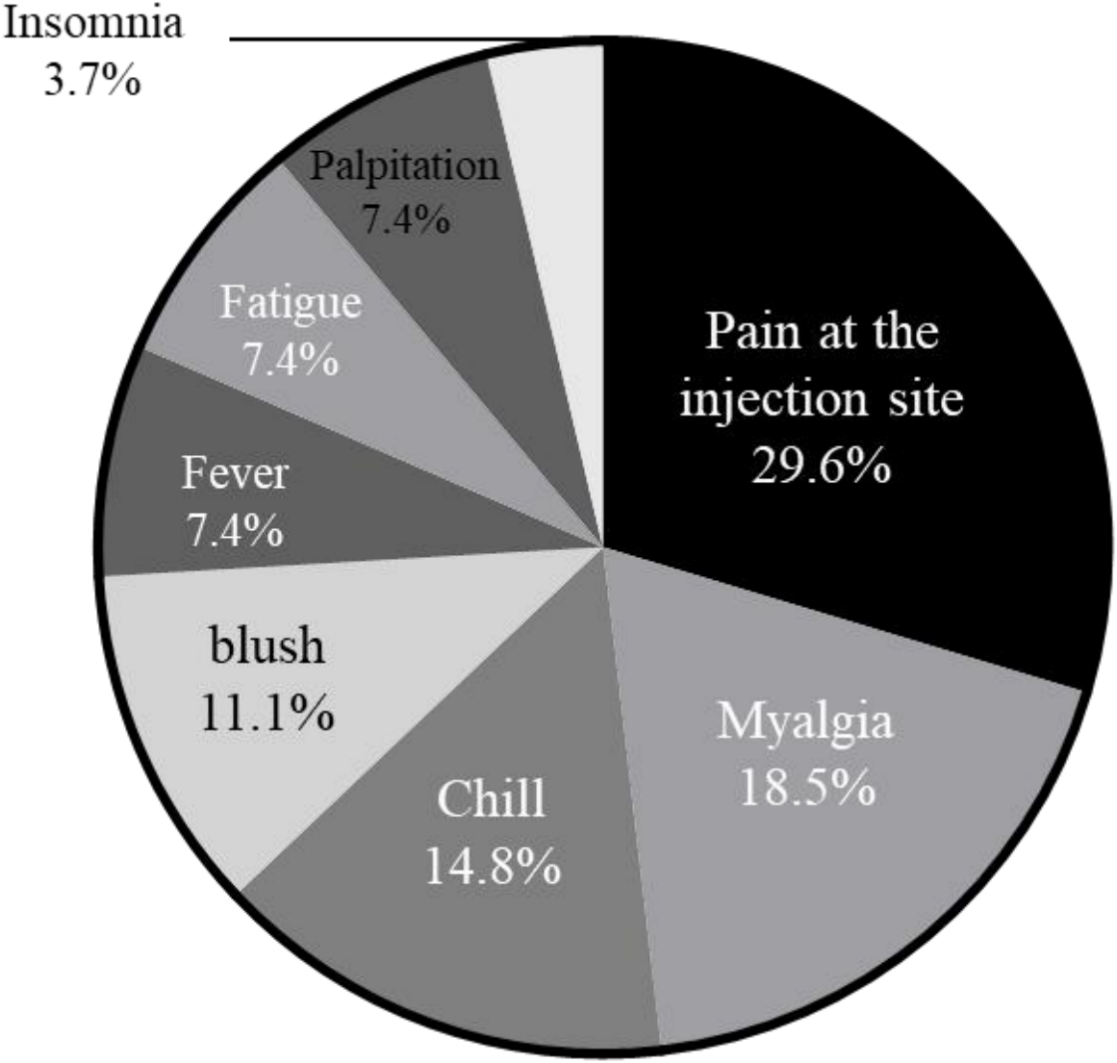
Side effects reported by PH children patients after COVID-19 vaccination.

12 PH children (16.4%) were vaccinated against COVID-19. There are 2 types of vaccination, namely Beijing Biotech (66.7%) and Beijing Sinovac vaccine (33.3%), which are both inactivated vaccines. 66.7% of vaccinated PH children reported at least one post-vaccination AEs. The most common AEs were local injection pain (29.6%), myalgia (18.5%), chills (14.8%), fever (7.4%) and fatigue (7.4%). 3 patients developed bruising or flushing at the vaccination site after vaccination. 2 patients developed palpitations after vaccination. Only 1 patient experienced insomnia after vaccination. No serious AEs were detected. The number and frequency of AEs of PH children after vaccination are shown in Figure 2.

### 3.4. COVID-19 prevalence, symptoms and severity

**Figure 3:**
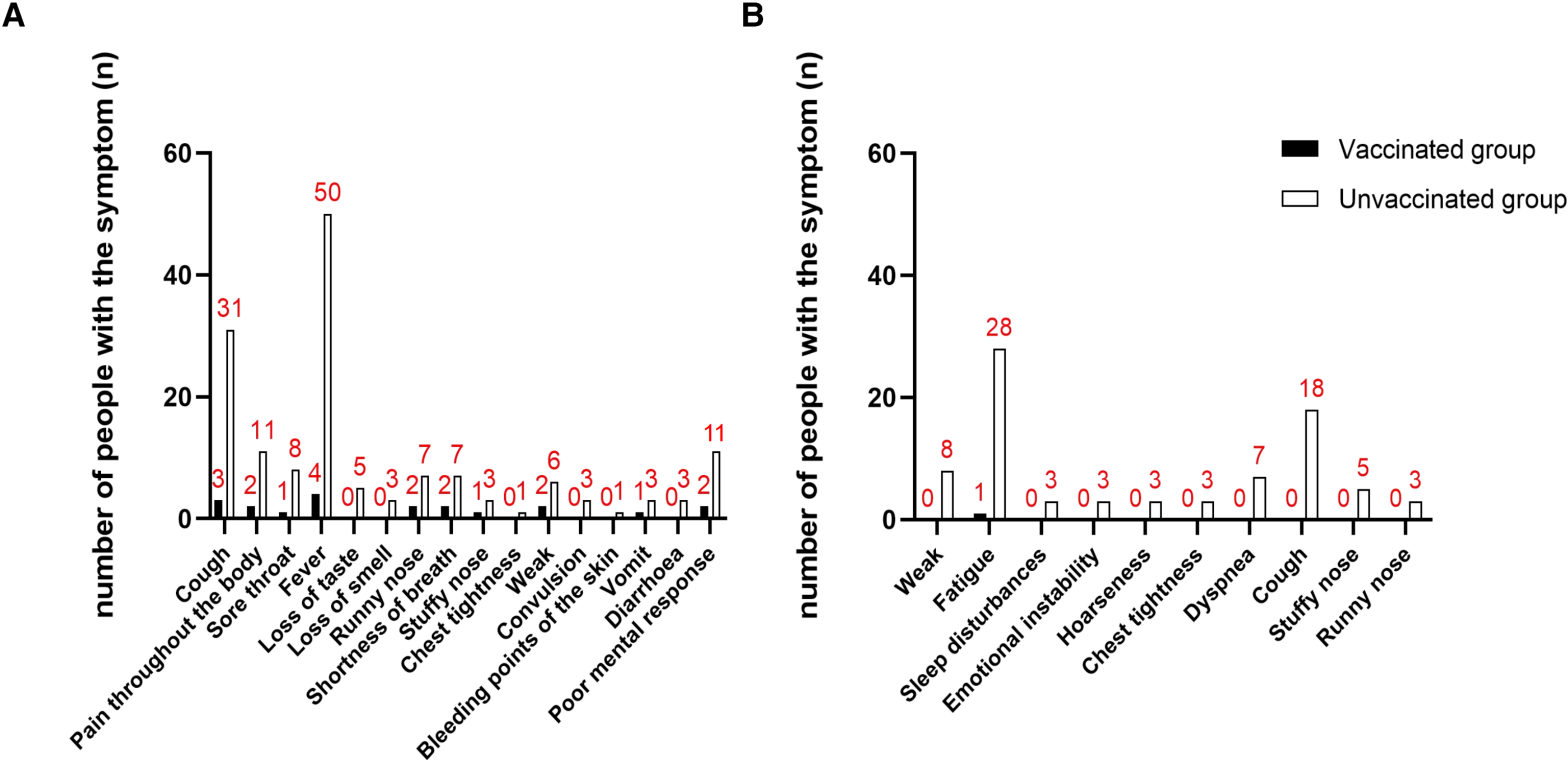
Distribution of COVID-infection symptoms of vaccinated and unvaccinated PH children. (**A**) Distribution of COVID-infection symptoms during infection in vaccinated and unvaccinated PH children; (**B**) distribution of COVID-infection symptoms during convalescence in vaccinated and unvaccinated PH children.

The prevalence of COVID-19 disease in PH children was lower in the vaccinated group than in the non-vaccinated group (vaccinated group: 5 cases, 41.7%; Non-vaccination group: 50 cases, 82.0%; RR = 0.51; *P* = 0.009; Supplementary Table). However, there was no significant difference in the duration (*P* = 0.898) and severity (*P* = 0.434) of COVID-19 disease between the two groups. The proportion of PH children in the vaccinated group with MISC (*P* = 0.762) and myocardial injury (*P* = 0.558) was also not lower than that in the unvaccinated group. Patients were divided into 3 classes according to the number of symptoms presented. There was no significant difference in the number of symptoms of COVID-19 disease between the vaccinated and non-vaccinated groups (*P* = 0.399). There was also no statistically significant difference in the distribution of symptoms of COVID-19 disease between the two groups (Supplementary Table). The most common symptoms of PH children infected with COVID-19 disease were fever (*P* = 0.09) and cough (*P* = 0.693). The statistical results of the remaining symptoms are shown in Supplementary Table and Figure 3A.

The most common symptom in PH children 1 month after COVID-19 nucleic acid or antigen test negative was fatigue (vaccination group: 1 case, 20.0%; non-vaccination group: 28 cases, 56.0%; *P* = 0.286) and cough (vaccinated group: 0 cases, 0.0%; Non-vaccination group: 18 cases, 36.0%; *P* = 0.160). Patients were divided into 3 classes according to the number of symptoms. 1 month after negative nucleic acid test or negative antigen test, PH children in the vaccinated group had fewer symptoms than those in the unvaccinated group (*P* = 0.049, Figure 3B and Table 3).

**Table 3:** Post-COVID-19 conditions reported by PH children patients or parents in vaccinated and unvaccinated groups.

	Total study group *n* (%)	Vaccinated *n* (%)	Unvaccinated *n* (%)	*P*-value
Number of symptoms in convalescence				0.049*
0	36 (72.0)	4 (80.0)	14 (28.0)	
1	5 (10.0)	1 (20.0)	15 (30.0)	
≥2	4 (8.0)	0 (0)	21 (42.0)	
Symptoms				
Weak	8 (14.5)	0 (0)	8 (16.0)	>0.999
Fatigue	29 (52.7)	1 (20.0)	28 (56.0)	0.286
Sleep disturbances	3 (5.5)	0 (0)	3 (6.0)	>0.999
Emotional instability	3 (5.5)	0 (0)	3 (6.0)	>0.999
Hoarseness	3 (5.5)	0 (0)	3 (6.0)	>0.999
Chest tightness	3 (5.5)	0 (0)	3 (6.0)	>0.999
Dyspnea	7 (12.7)	0 (0)	7 (14.0)	>0.999
Cough	18 (32.7)	0 (0)	18 (36.0)	0.160
Stuffy nose	5 (9.1)	0 (0)	5 (10.0)	>0.999
Runny nose	3 (5.5)	0 (0)	3 (6.0)	>0.999

* Chi-square test, *P* < 0.05.

## 4. Discussion

To our best knowledge, this study describes for the first time the reasons why PH children are reluctant to receive COVID-19 vaccines, as well as the factors that influence the willingness of PH children to receive COVID-19 vaccines. This increases confidence in the vaccine for PH children and their families and helps promote the development of public health policies related to vaccination.

The reasons for opting against the COVID-19 vaccination are diverse. The most common reason was fear of deterioration of pre-existing conditions. This is probabaly based on the fact that the childhood population has a weakened immune system due to the presence of hypoplasia of the immune system and comorbidities. The second common reason is not knowing the premises of the medical facility that provides the vaccination. This suggests that COVID-19 vaccine availability and advocacy should be increased. Another important reason is concern about AEs of vaccines. Similarly, in adults with PH or in children with BPD, fear of vaccine AEs is the most common reason for refusal to vaccinate against COVID-19 (20, 21). Therefore, when expanding vaccine policies, it is important to pay attention to promoting the confidence of vaccinated people in vaccines.

COVID-19 vaccination is safe in PH children without the presence of severe AEs. The presence of comorbidities can affect the severity of COVID-19 disease (3). Multiple studies have demonstrated the safety of COVID-19 vaccines in common or disease adult and child populations (14–16, 20). In the multivariate logistics regression model, PH children aged < 36 months and the presence of comorbidities was factors that prevented PH children from receiving COVID-19 vaccination. This is the same as the influencing factor of COVID-19 vaccination in adult PH (22). Other similar studies have also revealed the impact of factors such as parental education and household economic income on COVID-19 vaccination (20). Although our study did not address this, it gives direction to public health officials and future research to develop strategies. COVID-19 vaccination is safe and no serious AEs occur after COVID-19 vaccination. The most common AEs were pain at the vaccination site (8,66.7%), fever (7,58.3%), and fatigue (7,58.3%). None of the organ-specific damages, such as vascular damage or respiratory complications, occurred. Due to the use of inactivated vaccines, there were no breakthrough infectious events.

COVID-19 vaccination reduced the prevalence of COVID-19 disease. One study in February 2023 showed that the COVID-19 inactivated vaccine helped protect against symptomatic infection and halved the risk of moderate/severe disease in symptomatic patients (15). Booster doses of vaccines in the general population and in comorbid patients enhance protection against COVID-19 infection and COVID-19 disease severity (16). Although the prevalence of COVID-19 can be reduced, COVID-19 vaccination in PH children does not reduce the severity, number and distribution of symptoms in COVID-19 disease. Several studies have explored factors influencing the severity of COVID-19 disease (2, 23, 24). The most important influencing factor is the presence of severe comorbidities. However, of all the PH children included in this study, fewer had serious comorbidities, such as diabetes, hypertension, and immunodeficiency. On the other hand, none of the deaths in this study also reflect the mildness of the overall COVID-19 disease in the included PH children (25). In particular, PH children in the COVID-19 vaccine group had a reduction in the number of symptoms 1 month after a negative nucleic acid test or antigen test. This suggests a possible lag in the efficacy of COVID-19 vaccines. At the same time, the number of symptoms may also be related to the time point of follow-up and the population (26). 1/3 adults and 1/10 children still experience persistent sequelae after 1 year (26).

There are some advantages. First, the effectiveness and safety of COVID-19 vaccination was investigated in the population of children with PH. This increases confidence in the vaccine for children with PH and their families, which can promote willingness to vaccinate. Second, it reveals the most common reasons why children with PH and their families refuse to receive COVID-19 vaccines. This can help promote the development of public health policies related to vaccination.

There were several limitation in this study. First, this study is a single-center retrospective design and recall bias may occur. The subjects could be more representative if they were selected from more sources. Second, sample size should be larger which make the conclusion more reliable. Finally, socioeconomic factors of COVID-19 vaccination among children with PH should also be further into consideration, such as household income, educational attainment and health policy promotion.

## 5. Conclusions

In conclusion, The vaccination rate of COVID-19 vaccine is low in CHD-PAH and BPD-PH children while safe. The most common reason for refusing vaccinations in PH children is fear of exacerbation of existing disease. Vaccination against COVID-19 can reduce the proportion of COVID-19 disease and the number of uncomfortable symptoms after negative nucleic acid or antigen tests in PH children.

## Data Availability

The original contributions presented in the study are included in the article/**Supplementary Material**, further inquiries can be directed to the corresponding authors.
