# Supplementary material for: COVID-19 vaccination for children with pulmonary hypertension: efficacy, safety and reasons for opting against vaccination
Source: Front Pediatr. 2023 Oct 4;11:1259753. doi: 10.3389/fped.2023.1259753 (PMC10582704; doi:10.3389/fped.2023.1259753)
Supplement: Supplementary file 1 [file Table1.docx]

| Supplementary Table. COVID-19 infection, symptoms, and severity reported by PH children patients or parents in vaccinated and unvaccinated groups. | | | | |
| --- | --- | --- | --- | --- |
|  | Total Study Group  n (%) | Vaccinated n (%) | Unvaccinated n (%) | *P* Value |
| **COVID-19 disease** | 55 (75.3) | 5 (41.7) | 50 (82.0) | 0.009* |
| **Duration of COVID-19** | 7.0 (7.0) | 7.0 (9.0) | 7.0 (6.0) | 0.898 |
| **Severity** |  |  |  | 0.434 |
| No hospitalization | 35 (63.6) | 4 (80.0) | 31 (62.0) |  |
| hospitalization without oxygen | 7 (12.7) | 1 (20.0) | 7 (14.0) |  |
| hospitalization with oxygen | 16 (29.1) | 0 (0) | 8 (16.0) |  |
| ICU | 4 (7.3) | 0 (0) | 4 (8.0) |  |
| **Number of symptoms** |  |  |  | 0.399 |
| ≤1 | 27.3 (23.6) | 3 (60.0) | 12 (24.0) |  |
| 2-4 | 33 (60.0) | 1 (20.0) | 32 (64.0) |  |
| ≥5 | 7 (12.7) | 1 (20.0) | 6 (12.0) |  |
| **Clinical classification** |  |  |  | 0.113 |
| Mild | 34 (61.8) | 1 (20.0) | 33 (66.0) |  |
| Moderate | 15 (27.3) | 3 (60.0) | 12 (24.0) |  |
| Severe | 4 (7.3) | 1 (20.0) | 3 (6.0) |  |
| Critical | 2 (3.6) | 0 (0) | 2 (4.0) |  |
| **Symptoms** |  |  |  |  |
| Cough | 34 (52.7) | 3 (60.0) | 31 (62.0) | 0.693 |
| Pain throughout the body | 13 (23.6) | 2 (40.0) | 11 (22.0) | 0.725 |
| Sore throat | 9 (16.4) | 1 (20.0) | 8 (16.0) | 0.687 |
| Fever | 54 (98.1) | 4 (80.0) | 50 (100.0) | 0.09 |
| Loss of taste | 5 (9.1) | 0 (0) | 5 (10.0) | >0.999 |
| Loss of smell | 3 (5.5) | 0 (0) | 3 (6.0) | >0.999 |
| Runny nose | 9 (16.4) | 2 (40.0) | 7 (14.0) | 0.387 |
| Shortness of breath | 9 (16.4) | 2 (40.0) | 7 (14.0) | 0.387 |
| Stuffy nose | 4 (7.3) | 1 (20.0) | 3 (6.0) | 0.806 |
| Chest tightness | 1 (1.9) | 0 (0) | 1 (2.0) | >0.999 |
| Weak | 8 (14.5) | 2 (40.0) | 6 (12.0) | 0.304 |
| Convulsion | 3 (5.5) | 0 (0) | 3 (6.0) | >0.999 |
| Bleeding points of the skin | 1 (1.9) | 0 (0) | 1 (2.0) | >0.999 |
| Vomit | 4 (7.3) | 1 (20.0) | 3 (6.0) | 0.806 |
| Diarrhoea | 3 (5.5) | 0 (0) | 3 (6.0) | >0.999 |
| Poor mental response | 13 (23.6) | 2 (40.0) | 11 (22.0) | 0.725 |
| **MISC** | 8 (14.5) | 1 (20.0) | 7 (14.0) | 0.762 |
| **Pulmonary imaging changes** | 11 (20.0) | 3 (60.0) | 8 (16.0) | 0.079 |
| **Abnormal blood count** | 12 (21.8) | 3 (60.0) | 9 (18.0) | 0.110 |
| **Myocardial damage** | 11 (20.0) | 2 (40.0) | 9 (18.0) | 0.558 |
| **Increased HR** | 43 (78.2) | 4 (80.0) | 39 (78.0) | 1.000 |
| **Decreased oxygen saturation** | 29 (52.7) | 2 (40.0) | 27 (54.0) | 0.898 |
| MISC: Multisystem inflammatory syndrome – children, HR: Heart rate, * Chi-square correction test. | | | | |
